# The Rise of Data Politics: Digital China and the World

**DOI:** 10.1007/s12116-021-09319-8

**Published:** 2021-03-19

**Authors:** Lizhi Liu

**Affiliations:** McDonough School of Business, Georgetown University, Washington, DC USA

**Keywords:** China, Data, Politics of Technology, Privacy, Surveillance

## Abstract

Data has become one of the most valuable assets for governments and firms. Yet, we still have a limited understanding of how data reshapes international economic relations. This paper explores various aspects of data politics through the lens of China’s digital rise and the country’s global engagement. I start with the theoretical premise that data differs from traditional strategic assets (e.g., land, oil, and labor), in that it is nonrival and partially excludable. These characteristics have generated externality, commitment, and valuation problems, triggering three fundamental changes in China’s external economic relations. First, data’s *externality* problem makes it necessary for states to regulate data or even to pursue data sovereignty. However, clashes over data sovereignty can ignite conflicts between China and other countries. Second, the *commitment* problem in data use raises global concerns about foreign government surveillance. As data is easier to transfer across borders than physical commodities, Chinese tech companies’ investments abroad are vulnerable to national security investigations by foreign regulators. Chinese tech companies, therefore, confront a “deep versus broad” dilemma: deep ties with the Chinese government help promote their domestic business but jeopardize their international expansion. Lastly, data’s *valuation* problem makes traditional measures (e.g., GDP) ill-suited to measure the relative strengths of the world’s economies, which may distort perceptions of China and other states.

## Introduction

A 2017 *Economist* article asserted, “The world’s most valuable resource is no longer oil, but data.”^1^ Since then, world leaders, including Abe, Merkel, Modi, and Xi, have expressed similar ideas, reflecting the rising consensus on the strategic importance of data.^2^ Data is vital not only for its economic value but also for its growing influence on politics. For instance, Cambridge Analytica’s illicit harvesting of Facebook data reportedly influenced voting in the 2016 US presidential election, as well as the Brexit referendum in the UK earlier that year.^3^ The Trump administration also frequently cited data to defend the ban on Chinese tech giant Huawei, claiming that using Huawei’s 5G network would expose sensitive US data to the Chinese government.^4^

Despite its looming prominence, data politics remains vastly understudied. Data politics concerns the strategic interactions between sovereign states or between the state and non-state actors over the collection, processing, transfer, sale, or use of data. It constitutes a subset of digital politics, a much broader concept that also covers the political dynamics related to digital technologies, algorithms, and network security. Prior research has explored many facets of digital politics, such as censorship (Roberts 2018; King et al. 2013), online institutions (Liu 2018; Liu and Weingast 2018; Liu and Weingast 2020), privacy (Farrell and Newman 2019), and surveillance (Zuboff 2019; Greitens 2020; Xu 2020). Yet with very few exceptions,^5^ prior studies mostly focus on digital technologies rather than the data itself, which has overlooked how data’s unique features generate new political problems.

This paper aims to fill these gaps in the literature by exploring an important dimension of data politics, the *international politics of personal data*. To do the analysis, I focus on the context of China’s digital transformation and global engagement. China serves as a good analytical case for this study for two main reasons. First, China’s mass adoption of digital technologies has made data the lifeblood of every aspect of society. The government quickly adopted a national big data strategy and embraced the concept of “data sovereignty.” Second, China has become a global investor in the digital sector, which not only enables digital connectivity but also provokes geopolitical controversies worldwide.

The paper explores how the proliferation of data as a new strategic asset affects China’s engagement with the world, which fits the “China in the world” paradigm (Fravel et al. 2020). I first theorize how data differs from traditional assets (e.g., land and oil) in terms of economic characteristics and why this distinction makes data politics worth researching. Drawing on the nascent literature on the economics of data, I show that data is a (1) *nonrival* (i.e., data can be infinitely used) and (2) *partially excludable* (i.e., it is not always possible to exclude nonpaying individuals from having access to data) good.

These characteristics—nonrivalry and partial excludability—generate three problems: externality, commitment, and valuation, all of which lay the foundation of data politics in China’s engagement with the world.

The first problem is *externality*. As data is nonrival and only partially excludable, it is a quasi-public good. In other words, data from those who choose to share it can generate benefits or costs for non-sharers. To balance the positive and negative externalities of data, China has joined many states in pursuing “data sovereignty” to regulate cross-border data flows. Clashes over data sovereignty—for example, China’s data localization requirements and US extraterritorial access to data—can generate conflicts between governments. These data regulations also constitute a new form of nontariff trade barrier for multinationals, which I illustrate by studying two cases in detail: (1) how China’s Cybersecurity Law affected Apple Inc. (2) and how Europe’s General Data Protection Regulation (GDPR) influenced Mobike, a Chinese bike-sharing start-up.

The second challenge is the *commitment* problem in data use and sharing. As data is only partially excludable and never perishes, there is great uncertainty regarding its future use. All tech companies that collect personal data face a commitment problem: how to guarantee that they will not disclose their user data to the government, which may abuse the data for surveillance or coercion. To attenuate this commitment problem, tech firms need to signal their independence from the government. Chinese tech firms suffer from such a commitment problem in overseas markets, in which they are constantly suspected of sharing foreign citizens’ data with the Chinese government. Being a private company does not help assuage such doubts. As a result, Chinese tech firms confront a “deep versus broad” dilemma: deep ties with the Chinese government reduce *domestic* political risks but raise *overseas* regulatory risks. I analyze the US investigation into TikTok to illustrate the “deep versus broad” dilemma and to show that TikTok has adopted a “costly signaling” strategy to attenuate the problem.

Lastly, data has a *valuation* problem. It is difficult to assess the price of data properly because it is nonrival and can generate new value through combining different datasets. The most commonly used measure of a country’s economy, gross domestic product (GDP), does not fully capture the value of data and many digital products that are free to use. This generates measurement errors in evaluations of a country’s economic strength, leading to perception distortions. To illustrate this point, I examine a case of China’s rural e-commerce initiative to explain the difficulty in quantifying the welfare gains from digital services.

The paper proceeds as follows. I first present background information on China’s digital transformation, outward foreign direct investment (FDI) in data-related sectors, and the recent policy shift toward data regulations. Then, I analyze how the economic features of data—nonrivalry and partial excludability—lead to externality, commitment, and valuation problems. I elaborate these three problems, discuss them in the Chinese context, and examine how they affect politics. I conclude by highlighting the growing prominence of data politics over time and beyond China.

## Background: Digital China and the Proliferation of Data

Digitization has transformed all aspects of urban China during the last decade. In 2006, the country’s online retail sales were merely 3% of the US sales. China now hosts the world’s largest e-commerce retail market, with a 40% share of global sales. Every day, 1 billion active users on WeChat exchange an average of 45 billion messages. Mobile pay has taken the country by storm: even beggars are accepting alms through QR codes. China has also established the world’s first official digital currency, as well as the first cyber court, where defendants, plaintiffs, and judges meet over video conferences. This fast-paced digitization process has launched an explosion of tech firms and startups. By market capitalization, Alibaba and Tencent were ranked in the top 10 publicly traded corporations globally as of early 2020. According to a report in 2018, China is also home to nine of the top 20 global internet firms by public or private market value; the USA hosts the rest. 6

Such domestic success has driven the Chinese digital sector to go global. In 2014–2016, China-based venture capital funds accounted for 14% of worldwide venture capital investment outside China, of which 75% invested in digital-related sectors such as artificial intelligence (AI), cloud computing, and digital payments (Woetzel et al. 2017). Chinese tech titans have also expanded their domestic rivalry to the global level through mergers and acquisitions. For example, two competing Southeast Asian online trading platforms, Lazada and Shopee, are backed by Alibaba and Tencent, respectively. Moreover, the Chinese government has been keen to export digital capacity. The Digital Silk Road, part of the Belt and Road Initiative, aims to facilitate global digital trade and to further strengthen China’s technology power and influence.

At home and abroad, data serves as the lifeblood of China’s digital transformation. Historically, data has always been valuable, for example, to reduce information asymmetry in economic transactions. Nevertheless, data did not play such a central role until the development of digital technologies that have enabled the mass collection, storage, and analysis of data. China produced approximately 7.8 trillion gigabytes (GB) of data in 2018, and this number is expected to reach 48.6 trillion GB by 2025, surpassing the USA.^7^ China’s large quantity of data may also constitute a competitive advantage in the age of AI, as “Data is what makes AI go; a very good scientist with a ton of data will beat a super scientist with a modest amount of data” (Lee 2018).

The Chinese government has long realized the strategic value of data. In November 2013, the National Bureau of Statistics signed agreements of strategic collaboration with 11 internet firms, incorporating the use of big data into government statistics. In 2014, China elevated the status of big data to a national strategy; action plans were released in 2015 that included the construction of a massive national data center in Guizhou Province. To accelerate the implementation of the big data strategy, President Xi led a Politburo study session in 2017, urging to “strengthen the ability to protect the nation’s crucial data resources, speed up relevant legislation, and improve protection of data property rights.”^8^ In April 2020, an important policy document listed data as one of the five basic factors of production, alongside land, labor, capital, and technology.^9^ This goes along with China’s 2020 “New Infrastructure” campaign, which intensively focuses on enhancing digital infrastructures, such as 5G networks, data centers, and AI.

China is hardly the only country that has registered the proliferation of data or acts on the policy front. By 2014, trans-border data flows accounted for roughly 3.5% of the global GDP, almost as valuable as the global trade in physical goods and services (Bughin and Lund 2017). How does the proliferation of data affect global politics?

## The Nature of Data and the Foundations of Data Politics

To examine data politics, we first need to discern the economic nature of data and, in particular, how data differs from other economic inputs.

Data is a strategic asset, but so are many other economic factors. Past studies have extensively explored the global politics involved in the ownership and trade of traditional goods, such as oil and land. One may attempt to apply existing theories on traditional goods to data.

Yet, data inherently differs from physical assets. Thus, data politics rests on a unique foundation. As noted above, a small but burgeoning literature in economics suggests that data has two characteristics that distinguish it from many other economic goods: nonrivalry (Jones and Tonetti 2019; Varian 2018) and partial excludability (Carrière-Swallow and Haksar 2019)*.*

*Nonrivalry* is a central feature of data (Jones and Tonetti 2019); it refers to the fact that data can be used an infinite number of times and by many parties simultaneously. For instance, different  governments and firms can collect the same set of personal data and citizens who give out their data still have access to the same amount of data. By contrast, rival inputs such as capital, oil, and labor do not replenish once used: when one consumes a barrel of oil, it reduces the availability of oil to others.

It is important to distinguish data from information and idea, which are also nonrival. I follow Jones and Tonetti (2019: 3), who define information as “all economic goods that are nonrival.” Information consists of two mutually exclusive parts: data and idea. Idea is the method of making an economic good (Romer 1990), whereas data is a factor of production (i.e., an input). For example, for a self-driving car, the idea is the machine-learning algorithm that guides the car, and the data is the input to produce the idea (Jones and Tonetti 2019).

Nonrivalry means that data can exhibit *increasing returns to scale* (Agrawal et al. 2018). Firms that use data can benefit from a “data feedback loop” (Farboodi et al. 2019) or direct network externalities (Goldfarb and Trefler 2018), in which a firm’s success attracts more users and user data, which improves the quality of products through AI and leads yet more users and data. Data can also yield increasing returns when combined with other factors of production. For instance, to double the production of Tesla automobiles, one needs to double the rival inputs, such as iron, used to manufacture them. However, the same data that empowers the self-driving algorithms in one Tesla can be used in two, three, or millions of cars.

Data’s nonrivalry feature, coupled with increasing returns to scale, has two economic implications. First, there may be substantial social gains if data is widely shared across firms and countries. Second, if data is not broadly shared, the quantity held by a firm or country can generate a competitive advantage. Bigger firms and countries that are associated with larger quantities of data can be more productive in the digital era, leading to greater market concentration (Table 1).

**Table 1:**
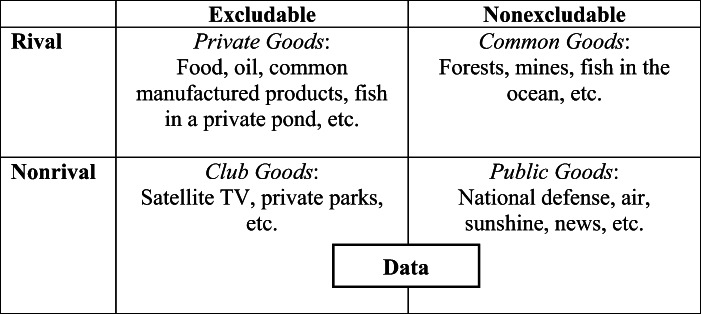
Types of economic goods based on rivalry and excludability

Data is also *partially excludable*. Excludability concerns whether it is possible to prevent nonpaying individuals from consuming certain products. Traditional economic inputs are often excludable. For instance, a state can prevent individuals from occupying territory or consuming oil if they have not paid for it. However, it is not always possible to block nonpaying individuals’ access to data.

Data is partially excludable because it is easy and inexpensive to duplicate and to transfer over a long distance, even across a country’s border. This generates a high risk for unauthorized sales, usage, or theft of data. Of course, data can still be excludable if it is fully encrypted, anonymized, or stored offline (Jones and Tonetti 2019). However, encryption, anonymization, and offline isolation involve a cost and can make the data less valuable. Many digital products, such as cloud computing and GPS navigation, can no longer function without real-time data sharing.

Note that the above discussion about excludability focuses on the general feature of data. Nevertheless, data’s excludability can vary depending on context. For example, Newman (2010) showed that national data regulations could alter the excludability and distribution of data. Firms with significant data see data as a private asset and support restrictive laws on information access and sharing. Contrarily, firms with little data advocate a liberal environment for data sharing and usage. For simplicity, in this paper, I do not delve into these nuances, and my analysis focuses on personal data only.

As previewed above, the economic characteristics of data—nonrivalry and partial excludability—lead to three types of problems that lay the foundation for cross-national politics of personal data: externality, commitment, and valuation problems.

## Data’s Externality Problems and China’s Move Toward Data Sovereignty

### Externality Problems of Data

Data’s partial excludability and nonrivalry features make it resemble a public good. It has both positive and negative externalities (Acquisti et al. 2016; Choi et al. 2019).

The sharing and creation of data can have positive externalities, such as beneficial spillovers on a third party. For instance, the collection of personal data from data sharers can train a machine-learning algorithm to improve the quality of service provided to others, including non-sharers. However, data also has negative externalities, especially on privacy. In some circumstances, data about the sharers can reveal some characteristics about non-sharers (MacCarthy 2011; Acemoglu et al. 2017). Erlich et al. (2018) found that their model only needed 2% of the target population’s genetic data to identify almost everyone, including those who had never undergone genetic testing. Likewise, a famous project conducted by MIT students found it easy to predict whether someone is gay based solely on the reported sexuality of their Facebook friends, leading to unintentional “outing” (Jernigan and Mistree 2009). Besides privacy concerns, mass digitization, and data-driven governance can generate other negative externalities. For example, fake data, rumors, and extremist opinions can be amplified through social media “echo chambers,” stirring social unrest and political polarization. Machine-learning algorithms can pick up existing stereotypes in data, making discriminatory acts perpetuate at an even larger scale.

Therefore, governments find it crucial to balance the positive and negative externalities of data, especially when the externalities come from *international data flows*. The cross-national exchange of data can lead to huge trade benefits but exposes citizen privacy to foreign surveillance, hacking, and data breaches. To regulate data’s externalities, market solutions are often not sufficient. For example, fearing the creative destruction caused by data sharing, firms can hoard data for themselves (Jones and Tonetti 2019). This limits the positive externalities and welfare gains data can generate. In addition, firms have an incentive to overcollect data but underinvest in data security and privacy protection (Carrière-Swallow and Haksar 2019; Jin 2018), which amplifies the negative externalities of data.

As a result, many governments have begun to claim data sovereignty to regulate trans-border data flows. An important reason for such a move is to foster positive externalities and contain negative ones.^10^ In the past few decades, many countries have established data protection laws or privacy regulations (Newman 2010). China recently followed suit. The rest of the section discusses the Chinese approach to data sovereignty and how the emergence of national digital borders affects tech firms within China and Chinese firms around the world.

### Data Sovereignty, Chinese Style

The concept of data sovereignty means that data should be subject to the laws and regulations of the nation-state in which it is generated and processed. It is a political effort to restrict data services along national borders. China’s data sovereignty is intricately linked to “cyber sovereignty,” a broader concept indicating that states should have control over the digital technologies, content, and infrastructures within their jurisdictions, which President Xi called for in 2015.^11^

To establish data sovereignty, China has issued four key documents to regulate trans-border data flows. The foundation was the Cybersecurity Law (网络安全法), which was enacted in 2016 and came into effect in 2017. The central government then issued three additional documents to implement the law: the Draft Measures on Security Assessment of the Cross-border Transfer of Personal Information of 2019 (个人信息出境安全评估办法), the Draft Measures for Data Security Management of 2019 (数据安全管理办法), and the Draft Information Security Technology-Guidelines for Data Cross-Border Transfer Security Assessment of 2017 (信息安全技术数据出境安全评估指南(草案)).

These documents comprise the core of China’s approach to data sovereignty: the requirement of data localization. The key is “local storage, outbound assessment” (Liu 2020). It requires all businesses operating in China to store select data (e.g., Chinese citizens’ personal data) on servers within China. If it is necessary to transfer locally stored data to another country, the Chinese government conducts a security assessment.^12^ The law also allows the Chinese government to conduct spot-checks on foreign businesses.^13^

While many other countries required data localization (Chander and Lê 2015) or struggled to maintain data sovereignty, China’s path has its own characteristics. So far, its data localization requirement is comprehensive but vague.^14^ Initiated in 2017, this requirement covered not only personal data but also important data related to critical information infrastructures. “Important data” is vaguely defined and can include any data, subject to the whims of the authorities.^15^

Additionally, the Chinese approach to data regulation is state centered, with “a unique combination of data protection and the government’s control over data flows.”^16^ The state has begun to regulate businesses to protect citizens from the unnecessary collection of personal data  for commercial purposes. For instance, in May 2020, the government fined a gym that illegally collected facial images of users.^17^ Meanwhile, the state itself has wide access to citizen data.

This state-centered model makes China different from the European Union and the USA, where the data regulations are citizen-based and market-based, respectively. As most of the digital platforms that collect EU citizen data are non-EU firms (e.g., Facebook, Google, and Apple), Europe has offered regulatory options to individual users, empowering them through the robust GDPR. The USA, however, contains the world’s most competitive digital companies and has largely relied on corporate self-regulation to govern data. While California recently adopted the California Consumer Privacy Act, which resembles GDPR, there is no federal-level privacy law. Compared with Europe, China’s data regulations focus more on protecting national security rather than citizen privacy. Furthermore, unlike the USA, the Chinese national government is very active in setting the agenda for data governance. 18

### The Inconvenient Consequences of Data Sovereignty

As China quickly erects borders for data, many countries have followed suit. The emergence of national digital borders will have at least four far-reaching political effects on China. First, China’s pursuit of data sovereignty will further strengthen digital authoritarianism on top of its already-sophisticated system of censorship and surveillance. As Chander and Lê (2015) argue, while censorship aims to keep information *outside* a country, data localization keeps all the citizen data *within* a country. This combination of censorship and data localization can lead to total control, further increasing the government’s ability to coerce and surveil.

Second, clashes over data sovereignty may become a new conflict hotspot for China and other countries. Many governments have erected data regulations with uncommon swiftness and little coordination, leading to incompatible rules. For instance, China’s data localization requirement could clash with the US government’s extraterritorial access to data. In 2018, the US Congress enacted the Clarifying Lawful Overseas Use of Data (CLOUD) Act, which authorizes law enforcement agencies to compel US-based tech firms (e.g., email and cloud service providers, such as Amazon and Microsoft) to hand over data—whether it is stored on US or foreign servers.^19^ The Act raises tricky questions about enforcement. An op-ed by a former US federal prosecutor discussed a hypothetical scenario, “What if a ‘wannabe America’s Toughest Sheriff’ serves a warrant on the Silicon Valley office of one of China’s major service providers to demand a Chinese official’s correspondence with party leaders stored in a Beijing server?”^20^ In this way, data rules can generate conflicts that require diplomatic resolution.

Third, China’s domestic legislation can generate problems when Chinese firms invest in countries with strict data regulations. Although China has no equivalent to the US CLOUD Act,^21^ its National Intelligence Law of 2017 sets broad requirements on organizations to “support, assist, and cooperate with the state intelligence work.”^22^ This law is often cited in the security debate regarding whether Huawei presents an espionage threat to the West. Huawei critics interpret the law as a legal foundation to grant the Chinese government access to the company’s data, even in the overseas market where data should be stored locally.^23^

Lastly, the emergence of national digital boarders constitutes a new trade barrier. Such borders will incur huge compliance costs for multinational tech firms, disrupting the global value chain and compromising economies of scale. Below, I examine two cases to demonstrate this point: Apple in China and Mobike in Europe. They respectively demonstrate how the Chinese Cybersecurity Law affects multinational operations within China and how data regulations elsewhere penalize Chinese businesses in overseas markets.

### Case I: Apple in China

China has been a crucial market for Apple Inc.—the second-largest market for iPhone sales and the key manufacturing base—but Apple’s relationship with China has never been easy.

Many of Apple’s skirmishes with Beijing have centered on user data. The company has frequently come under fire from China’s state media, which suspected that Apple had provided user information to US intelligence agencies. In 2014, China’s state broadcaster accused Apple of posing a national security threat. It expressed concerns over iPhone’s “Frequent Locations” feature, which tracks real-time user locations for Apple apps, such as Weather and Maps. The state media claimed that the location data was extremely sensitive, as it could reveal China’s economic conditions and “even state secrets.”^24^

In response, Apple explained that location data was encrypted and stored on individual phones, rather than on company servers. The company also denied helping the US government spy on iPhone users.^25^ To assure the Chinese government, Apple began to store some Chinese user data on servers in mainland China. The data was moved from overseas to a domestic plant managed by the state-owned enterprise (SOE) China Telecom.

In 2016, China issued the Cybersecurity Law, which requires all online data of Chinese citizens to be stored on domestic servers. The official purpose of the law is to help combat rising terrorism and hacking. Nevertheless, critics worry the law may strengthen the censorship regime and raise barriers to entry for foreign firms, because foreign firms will face higher adjustment costs under the law.^26^

Apple chose to comply. In 2017, it started to build a new data center operated by a company cofounded by the Guizhou provincial government. In 2018, Apple relocated to China the keys needed to unlock Chinese users’ iCloud accounts.^27^ This action provoked considerable criticism from human rights activists in the West.^28^

Apple’s situation reflects a dilemma common to multinationals: not complying with Chinese data regulations risks a crackdown from the Chinese government, whereas compliance creates a liability in Western markets.

### Case II: Mobike in Europe

Mobike is one of China’s most renowned startups. It used to be the world’s largest bike-sharing operator, allowing users to find a bike via an app, unlock it with a single keystroke, and park it anywhere in the city when they are done. Founded in 2015, the startup quickly gained popularity in China. In 2017, Mobike launched its European operations. By 2018, it had deployed 200,000 bicycles in 23 cities across Europe, accumulating 200 million registered users worldwide.

Mobike’s real value lies not in the bicycles it owns, but in the user data it collects via the app. Such information, if resold, can generate substantial revenue for the company. As investor Mark Wiseman commented, “The owners of those bicycles (i.e., Mobike) know where they are being ridden, when and by whom. … I’d be willing to pay a lot of money for that data.”^29^

The data-driven model—which is what makes Mobike successful—also leaves the company vulnerable to data regulations. In May 2018, shortly after Mobike entered the European market, the EU’s GDPR came into force. The world’s toughest data regulation regime, GDPR, aims to protect EU citizens’ personal data and privacy. Under GDPR, regulators can fine noncompliant companies up to 4% of their annual global turnover or €20 million, whichever is greater. In a prominent case, Google was fined $57 million in early 2019 for “lack of transparency, inadequate information and lack of valid consent regarding the ads personalization.”^30^

In late 2018, after establishing a presence in multiple cities, Mobike was reported to be under investigation by Germany’s privacy watchdog for a potential breach of GDPR. The details of the probe were not made public, but according to Swedish privacy expert Alexander Hanff, the regulator’s concerns were likely related to three aspects of Mobike’s operations in Europe.^31^

The first is the excessive collection of user data. The app collects a significant amount of data, including precise location data, even when the user is not using the app. Second, Mobike’s privacy policy allows it to share this data with third-party companies, and users have no control over the data handed over to these third parties. The third and biggest concern is that Mobike sends user data to China and Singapore, where it has its headquarters. This directly challenges GDPR, which imposes stringent restrictions on transfers of personal data outside the European Union.

Three months after the probe was disclosed, Mobike started seeking to spin off its European arm. It eventually withdrew from the European market after a management buyout in 2019,^32^ likely due, at least in part, to Europe’s strict data regulation.

## Commitment Problems and the “Deep versus Broad” Dilemma for Chinese Firms

### Commitment Problems of Data

Data’s nonrivalry feature means that it can be reused infinitely. The reuse of data is difficult to detect because it is only partially excludable. Therefore, once citizens exchange their personal data for free services from digital firms, they usually lose control over future use of the data (Jin 2018).

This uncertainty regarding future data use is a critical political matter, because it can generate concerns about foreign government surveillance. An important premise of these concerns is that data is valuable and states have high incentives to control and compete for data. Concerns about foreign government surveillance arise from two commitment problems: (1) multinational firms cannot credibly commit to not sharing personal data with their home government; and (2) the home government cannot commit to not abusing personal data for surveillance or for other political purposes that encroach on individual liberty.

Both commitment problems are more severe in an authoritarian country such as China. Regarding problem (1), firms are less likely to defy data requests from an authoritarian government. In 2016, Apple openly resisted the FBI’s request to unlock the iPhone belonging to the shooter in a terrorist incident in San Bernardino, California. It is not impossible for firms to make such an open refusal in authoritarian states, but it is far riskier. For example, Russia banned the messenger app Telegram for 2 years after it refused to share encryption keys with Roskomnadzor.^33^ As for commitment problem (2), autocrats may have a greater incentive to engage in digital surveillance, as they face an information issue of not knowing their citizens’ true preferences, due to preference falsification (Xu 2020).

There are two important caveats regarding the commitment problems. First, firms and governments in democracies are not immune from these commitment problems. Government mass surveillance projects are on the rise globally, as demonstrated by the US NSA’s PRISM program, the British Tempora project, and many others. According to the annual transparency reports published by Facebook, Apple, and Google, a significant portion of government data requests come from democracies, and these tech giants have fulfilled the vast majority of these requests in recent years.^34^ Therefore, the commitment problem in data use is not unique to autocracies. One advantage for firms based in democracies, however, is that the institutional constraints—e.g., checks on a government’s arbitrary acts (including accessing data), free press, and the rule of law—are stronger in democracies. These constraints on both firms and governments can serve as commitment devices, albeit imperfect ones, to bind future actions of data abuse. Second, even for firms based in autocracies, that a firm cannot credibly commit *ex ante* does not mean it will surely hand user data to its home government *ex post*. Firms bear reputational costs of abusing their user data, and their interests are usually not aligned with the interests of political authorities.

When Chinese tech companies expand globally, they suffer from these commitment problems, which leave them vulnerable to national security investigations worldwide. As data is at the core of tech firms’ business operations, they must collect personal data of foreign users. A growing number of foreign governments, however, maintain that China-based tech firms may expose foreign citizen data to the Chinese government. Such distrust in Chinese tech firms is certainly related to—but not solely driven by—the rising skepticism toward China itself. The underlying issue is a general commitment problem facing every multinational tech firm and every government.

The rest of this section specifies how the commitment problem affects Chinese tech firms through a “deep versus broad” dilemma. I illustrate this point by examining the case study of the US investigation of TikTok.

### Ownership Bias and Blurred State–Business Boundaries

All governments seek to maximize the economic benefits, while minimizing national security risks associated with inward FDI. When dealing with Chinese investors, foreign governments in the past had “ownership bias”: they used to be more cautious with investments from Chinese SOEs than those from private firms. This is consistent with existing research on the Chinese corporate sector, which has overwhelmingly relied on firm ownership to demarcate the boundary of the state. SOEs are portrayed as vehicles for the state to fulfill domestic political goals and to exert economic statecraft globally. By contrast, private enterprises are excluded from, or only peripherally connected to, the political process.^35^ Although private firms are not entirely autonomous from the state, they are not as connected to the state as SOEs.

However, ownership has long been a fuzzy concept in China. In particular, the rise of tech companies has blurred the state–business boundary, making it porous, elusive, and fluid. Unlike traditional private companies that are marginalized in state initiatives, privately held Big Techs have taken many strategic roles that only SOEs could assume in the past.

Within China, there are extensive collaborations between the state and digital companies over a wide range of issues. Underlying this is a phenomenon that I call *institutional outsourcing*^36^: as the authoritarian state is unable or unwilling to reform formal institutions, it has implicitly or explicitly outsourced some institutional functions to key private actors, particularly digital platforms (Liu 2018). To clarify, institutional outsourcing does not mean that these private Big Techs have become part of the state or act mainly upon the state’s political directives. Big Techs are still primarily profit-driven, pursuing commercial interests that are not fully aligned with the state’s goal. Institutional outsourcing features in the extensive collaborations between the state and Big Techs to solve various governance issues that the state falls short of addressing itself.

In the economic realm, online trading platforms assist the state to create market institutions (e.g., contract enforcement, fraud prevention), enforce laws (e.g., Alibaba and JD help the Supreme Court enforce debt repayment), conduct policy experiments (e.g., Alibaba helps local governments build “smart cities”), and facilitate rural development (Khanna et al. 2019; Koo and Eesley 2020; Couture et al. 2021).

In the political realm, private tech companies, such as social media platforms, help the state conduct surveillance and perform censorship (Gallagher and Miller 2018). In the social area, during the COVID-19 outbreak, Big Techs such as Alibaba and Tencent helped local governments in Hangzhou and Shenzhen build the “health code,” a contact-tracing app to contain the spread of the virus.

The intimate domestic collaboration between the Chinese state and private tech companies, especially on the data front, can become a liability for these firms in the overseas market. Although many of these collaborations are nonpolitical and Big Techs reportedly have turned down government data requests occasionally,^37^ the lack of institutionalized checks on the state’s arbitrary power complicates efforts by private techs to prove their independence. Overseas regulators no longer view private ownership as a credible sign of a firm’s relative autonomy from the Chinese state, which, for example, constitutes a major hurdle faced by the Chinese tech giant Huawei as it rolls out its 5G network worldwide.^38^

### Private Techs’ “Deep versus Broad” Dilemma in Global Expansion

As Chinese firms rapidly expand their business worldwide (Ratigan 2020), foreign governments hosting Chinese investments face an information problem. Not all private firms are agents of the Chinese state (Kastner and Pearson 2020), but foreign governments do not have complete information to identify the real type of a Chinese firm—regarding whether it will pose a national security threat. As firm ownership no longer suffices to predict firm type, foreign regulators tend to infer a Chinese firm’s type from its business activities within China.

Therefore, China-originated private firms increasingly confront a “deep versus broad” dilemma: deep embeddedness in the Chinese market—where it is necessary to build strong political connections—takes a toll on their global expansion. Herein lies the rub: a deep tie with the Chinese government reduces domestic political risks, but increases overseas regulatory risks.

This dilemma is particularly salient for Chinese firms operating in countries with strong anti-China sentiment, and exceptionally tricky for digital companies, which collect personal data for business operation. As discussed previously, data is nonrival and only partially excludable. These features make it difficult for the firm to commit ex ante to not share data with the Chinese government ex post. As data is easier to transfer across borders than physical commodities, Chinese tech firms are particularly vulnerable to national security investigations by foreign governments.

Is there a way to resolve, or at least attenuate, the dilemma? Some tech firms have engaged in costly signaling by building “walls” to separate their domestic markets from overseas businesses. In the following, I examine the US national security investigation into TikTok to explicate the logic of data politics: how the collection of personal data becomes a sensitive issue, how the firm’s inability to commit generates suspicion, and how TikTok seeks to address the commitment problem by costly signaling its independence from the Chinese government.

### Case: TikTok’s Commitment Problems and Costly Signaling

TikTok, owned by the Beijing-based tech giant ByteDance, is the first Chinese social app to take the world by storm.^39^ This AI-empowered platform allows users to create and share short videos, often of themselves dancing, lip-syncing, or cooking. By April 2020, a mere three years after its launch, the app hit 2 billion downloads worldwide, amassing 800 million monthly active users,^40^ 26.5 million of whom reside in the USA.^41^

TikTok’s meteoric rise has prompted US lawmakers to panic. Although it is privately owned and operates exclusively outside of China, it has been suspected of being under the control of the Chinese government. In an open letter released in October 2019, Senators Tom Cotton and Chuck Schumer contended that “TikTok is a potential counterintelligence threat we cannot ignore,” requesting intelligence officials to assess the national security risk posed by TikTok and other China-owned platforms.^42^ In November 2019, the US government launched a national security investigation into TikTok.^43^ As of March 2020, several US government agencies, including the Transportation Security Administration and the US Army, had banned the app from employee devices.^44^

How could an app famous for lip-syncing and dance challenges raise national security concerns? The investigation is not driven simply by the growing US–China rift that has politicized business matters. Data politics is also at play, which can emerge between any country dyad and regarding any multinational firm that collects personal data.^45^

The senators’ open letter specified two data-related concerns over TikTok. The first was how the company stores and handles US user data and whether such data will be exposed to the Chinese government. The second is related to the potential for disinformation and content manipulation. They argued that TikTok has reportedly censored political content disliked by the Chinese Communist Party, including material linked to the Hong Kong protests, Tibet, and Taiwan. They also mentioned that TikTok may serve as a potential forum for foreign meddling in US elections, as Facebook did in 2016.^46^

TikTok denied all of these charges. In a statement, it emphasized that all US consumer data is stored in the US and backed up in Singapore, arguing that “our data centers are located entirely outside of China, and none of our data is subject to Chinese law.” It also claimed that it would not censor content based on political sensitivities and was not influenced by any government.^47^

Some lawmakers disparaged TikTok’s denials as cheap talk. Senator Josh Hawley commented in a hearing that “TikTok claims they don’t store American user data in China. That’s nice. But all it takes is one knock on the door of their parent company, based in China, from a Communist Party official for that data to be transferred to the Chinese government’s hands, whenever they need it.”^48^

This reflects a commitment problem facing TikTok: the firm cannot credibly commit ex ante that it will not share user data with the Chinese government ex post. Although every firm has a similar commitment problem, firms from an authoritarian country suffer from it more because authoritarian countries do not have strong institutional constraints on the ruler’s arbitrary power. As the senators’ open letter claims, “without an independent judiciary to review requests made by the Chinese government for data or other actions, there is no legal mechanism for Chinese companies to appeal if they disagree with a request.”^49^

This commitment problem leads to the “deep versus broad” dilemma. TikTok’s parent company, ByteDance, is deeply embedded in the Chinese market. To some extent, ByteDance’s deep success within China is taking a toll on TikTok’s international expansion.

To expand globally, TikTok has to signal its independence from ByteDance’s China business. It made three costly moves to send this signal. First, TikTok limited its own revenue sources by not allowing any paid political advertising on its platform.^50^ Second, it started to look for global headquarters outside China, in Singapore, London, or Dublin.^51^ Third, TikTok announced that it would no longer use China-based moderators to monitor overseas content.^52^ ByteDance even prohibited its own China-based engineers from accessing TikTok’s US user data and code.^53^

These moves, however, were insufficient to address the commitment problem in the eyes of the Trump administration, which sought to propel ByteDance to sell TikTok. On August 6, President Trump issued an Executive Order banning all US transactions with ByteDance in 45 days, claiming that TikTok’s data collection “threatens to allow the Chinese Communist Party access to Americans’ personal and proprietary information.”^54^ On August 14, a follow-up Executive Order stated that ByteDance shall “divest all interests and rights” in TikTok’s US business and destroy the US user data collected by TikTok.^55^

Facing the ban, ByteDance reportedly negotiated with various interested parties in the USA over the sale of TikTok. However, the complication is that, to make the deal successful, ByteDance also needed to abide by China’s newly revised rule that restricts exports of recommendation algorithms and AI technologies. In other words, to satisfy both governments, TikTok needed to solve the commitment problem without transferring its core technologies.

In September, ByteDance reached a preliminary deal with two US companies, Oracle and Walmart. Tentatively approved by Trump, this deal points to a potential solution to the commitment problem. Under the deal, ByteDance will create a US-based subsidiary called TikTok Global, in which Oracle and Walmart, combined, have a 20% stake. TikTok Global will provide full TikTok services. To satisfy the national security requirements, US user data will be stored on Oracle’s cloud infrastructure, and Oracle will get full access to review TikTok’s source code and updates. ByteDance, on the other hand, neither needs to sell nor to transfer TikTok’s technologies and algorithms and will have an 80% share of TikTok Global. This deal has not been finalized by the time of writing, and it remains uncertain whether the deal can eventually address the commitment problem.

## Data’s Valuation Problems and the Measurement Bias on States’ Power

### Valuation Problems of Data

Data proliferates as an economic input in the modern economy, but its economic value is difficult to measure for two reasons. First, data is nonrival and intangible. It therefore cannot be depleted or worn in the way that many tangible goods are. Second, its value does not necessarily depreciate over time. Quite often, data fusion—the recombination of different independent datasets—generates new value (Li et al. 2019).

This valuation problem makes it challenging to assess a country’s real economic strength. With the rise of the digital economy, a country’s operation increasingly relies on the collection, exchange, and sale of data, for which we lack proper valuation. What particularly complicates the measurement problem is the prevalence of free digital products, such as search engines, social media, and email. As the use of digital products involves no or few monetary payments, their value is substantially underestimated by current measures of economic power, such as GDP.

The valuation of data is not simply an economic problem. It matters substantially in politics. Politicians often develop foreign policies based on the economic conditions of their own and foreign states. For a long time, states have used GDP and GDP growth to measure countries’ relative economic power. As data plays an increasingly important role in the world economy, incomplete measurements of countries’ economies, such as GDP, will produce inaccurate assessments of world powers and thus distorted foreign policies.

The rest of this section discusses the problems of the GDP measure in the digital era, and the difficulty of measuring the welfare generated by digital services, using China’s rural e-commerce project as an example. I also discuss how this measurement problem can distort our view of the relative strengths of states.

#### GDP’s Measurement Error in the Digital Era

Most political analyses use the GDP statistic to measure the size of an economy and states’ relative economic power. For example, China’s miraculous GDP growth has received much attention in popular media and research.

The rise of the digital economy, however, raises a cautionary note regarding the use of GDP to measure power. Prior research has questioned the reliability of China’s GDP figures, citing evidence of data manipulation in official statistics (e.g., Wallace 2016). But there is a deeper concern: by design, the GDP statistic cannot capture economic welfare, particularly the welfare gains from free or nearly free digital products. This problem is not unique to China; it is universal.

GDP measures the market value of all final products and services produced in an economy. It has two inherent limitations. First, it does not include zero-priced products in the market (Aitken 2019). Second, GDP primarily covers market production, which provides a clear set of quantities and prices. It does not include nonmarket production, such as household production for self-use (e.g., cooking and maintenance) or unpaid volunteer work (IMF 2018).

These measurement problems become increasingly thorny in the digital era. Internet services, such as Facebook and Google, are often free to use or strikingly underpriced. For example, the Chinese messaging app WeChat allows migrant workers to have free video calls with children they have left in their home villages. This welfare gain is not fully captured by the GDP measure, as WeChat users *do not pay* for the service. Instead, the users barter their personal data for such digital products, enabling the digital companies to monetize by selling targeted ads or reselling data. Although these advertising profits can be counted as part of the GDP, they represent only a fraction of the value of data (Brynjolfsson and Collis 2019). Therefore, many digital services have reduced the cost of living and improved the quality of citizens’ lives, yet the GDP measure underestimates their effects.

Another feature of the digital economy is that voluntary contributions—e.g., Wikipedia, its Chinese counterpart Baidu Baike, and other open-source software—generate substantial productivity. Again, GDP does not capture this part of the welfare gain. A good example shown by Brynjolfsson and Collis (2019) illustrates how the GDP measure can be *negatively* correlated with actual economic well-being: “Consider *Encyclopedia Britannica* and Wikipedia. Britannica used to cost several thousand dollars, meaning its customers considered it to be worth at least that amount. Wikipedia, a free service, has far more articles, at comparable quality, than Britannica ever did. Measured by consumer spending, the industry is shrinking. ... But measured by benefits, consumers have never been better off” (Brynjolfsson and Collis 2019: 145).

The following case of China’s rural e-commerce initiative reflects the difficulty of fully capturing the welfare gains from digital services and how consumption gains can take place without changes in output or nominal income.

#### Case: Quantifying Household Welfare Gain from Rural E-Commerce

Around 2014, most e-commerce activities in China still took place in urban areas, leading to a national policy initiative to expand e-commerce to rural areas. To implement this policy, the Chinese government partnered with a large online trading platform. The program aimed to introduce e-commerce into 100,000 villages by installing an e-commerce service point in each participating village. Each service point hired a terminal manager to help villagers buy and sell products on the platform. The firm and the government also connected the participating village with the urban center and fully subsidized the shipping costs of packaging going into and out of the village.

To study how e-commerce connectivity causally affects rural household welfare, Couture, Faber, Gu, and I conducted a field experiment in collaboration with the firm (Couture et al. 2021). We randomized the location of e-commerce service points across 100 villages in three provinces of China and found substantial consumption welfare gains for adopters of e-commerce. We found that e-commerce provided rural households with cheaper, more, and higher-quality products, as well as better shopping amenities. E-commerce thus raised household purchasing power by producing consumption-side welfare gains. However, the study did not find welfare gains on the production side. There was no evidence that e-commerce generated significant effects on nominal income or entrepreneurship activities, at least in the short to medium term.

This research demonstrated the difficulty of capturing the welfare changes brought by digital services. The shift from offline to online commerce led to many changes in product variety, quality, and convenience in shopping. To capture the associated welfare changes, we had to collect detailed information on every online/offline transaction each household had conducted, and on the products offered at the local physical stores. This led to a longitudinal survey of two rounds from 2800/3800 rural households and 11,500 price quotes from physical village stores. Measuring the welfare of other digital services will be even more complicated if it involves free services and new products. This is why the International Monetary Fund and national governments have called for new methods to measure the digital economy.^56^

#### How Data Valuation Problems Matter Politically

As data plays an increasingly important role as an economic input, GDP (per capita) can generate greater measurement error when used as a proxy for economic strength. Yet, why does this statistical problem matter politically?

Perceptions matter in China’s engagement with the world (Li 2020; Huang 2020). Having a disproportionate focus on GDP makes it difficult to accurately assess the relative strengths of world economies. It may distort Chinese perceptions of the outside world and foreign perceptions of Chinese economic resilience. For instance, Brynjolfsson et al. (2019) designed a new measure of economic power by incorporating the welfare contribution of free digital products, which they called GDP-B. Adding Facebook alone caused GDP-B to grow by 1.91% per year; by comparison, average real GDP growth over the same period was 1.83% per year. In other words, Facebook alone added 0.05–0.11% to GDP-B annual growth. The US growth rate has therefore been underestimated.

A look beyond GDP also helps examine the resilience of the Chinese regime. Many have taken the recent decline of GDP growth as a sign of weakness. It is puzzling that—despite the overwhelming consensus that the Communist Party relies on economic performance for legitimacy—China’s economic slowdown has not led to major political unrest. One plausible explanation is that China’s digital transformation has unleashed consumer welfare and generated stabilizing effects for the regime.

## Conclusion

As digital technologies transform how individuals work, trade, and live, they have also begun to influence how states interact. At the core of this change is the proliferation of data as a strategic asset. Many have equated data with oil to highlight the value of data. However, as this paper indicates, data differs from oil in nature, and data politics operates on unique foundations that are worth researching. Exploiting China as the analytical context, this paper provides an analytical framework to study data politics, showing how data’s externality, commitment, and valuation problems generate new conflicts in China’s external economic relations.

Lessons from China also shed light on international economic relations in general. As the global economy gets digitized, data has become a new frontier of geopolitical rivalry. For example, the European Union recently fined Google and Amazon $163 million for violating its privacy law, reflecting a concern about data’s negative externality. The Russian meddling in social media in the 2016 US election indicates that personal data can be weaponized to influence foreign states, which points to the commitment problem in data use. Moreover, over two dozen countries have imposed some form of digital tax by 2020, making tech companies pay their “fair” share of taxes, underlying which is data’s valuation problem.

At a deeper level, data politics is blossoming because data has changed the basis of power. A state’s strength lies in not only its military or trading power, but also its capacity to collect, refine, and utilize data. Data politics is at play, and it will only increase in salience over time.

## Footnotes

1 The Economist, “The world’s most valuable resource is no longer oil, but data,” May 6, 2017, https://www.economist.com/leaders/2017/05/06/the-worlds-most-valuable-resource-is-no-longer-oil-but-data

2 Nikkei Asia, “Trump and Xi trade barbs over 5G before summit,” June 29, 2019, https://asia.nikkei.com/Politics/G-20-summit-Osaka/Trump-and-Xi-trade-barbs-over-5G-before-summit and Harvard Kennedy School Belfer Center, “The Geopolitics of Information,” May 28, 2019, https://www.belfercenter.org/publication/geopolitics-information

3 The New Yorker, “New Evidence Emerges of Steve Bannon and Cambridge Analytica’s Role in Brexit,” November 18, 2018, https://www.newyorker.com/news/news-desk/new-evidence-emerges-of-steve-bannon-and-cambridge-analyticas-role-in-brexit

4 CNBC, “Trump official compares Huawei to ‘the Mafia’ as White House works on 5G battle plan,” February 25, 2020, https://www.cnbc.com/2020/02/25/trump-official-calls-huawei-mafia-as-white-house-works-on-5g-battle-plan.html

5 For example, Abraham Newman (2010) examined how a firm’s ownership of information assets shapes its policy preferences toward privacy regulations in developed markets.

6 MarketWatch, “China has 9 of the world’s 20 biggest tech companies,” May 31, 2018, https://www.marketwatch.com/story/china-has-9-of-the-worlds-20-biggest-tech-companies-2018-05-31

7 CNBC, “As information increasingly drives economies, China is set to overtake the US in race for data,” February 13, 2019, https://www.cnbc.com/2019/02/14/china-will-create-more-data-than-the-us-by-2025-idc-report.html

8 China Daily, “Xi Jinping: Implement the Big Data National Strategy to Accelerate the Construction of Digital China,” December 12, 2017, http://www.chinadaily.com.cn/interface/flipboard/1142846/2017-12-12/cd_35280418.html

9 See “Opinions of the Central Committee of the Communist Party of China and the State Council on Improving the Market-based Allocation Mechanism of Production Factors (中共中央国务院关于构建更加完善的要素市场化配置体制机制的意见),” March 30, 2020, http://www.gov.cn/zhengce/2020-04/09/content_5500622.htm

10 This does not mean that privacy externality is the only reason for states to assert data sovereignty. States are also driven by political considerations such as ensuring the strategic control over data. For example, as many developing countries lack indigenous technology capacities and privacy regulations, their citizens’ data may receive better protection if the data is stored in other countries where privacy norms are stronger. In this scenario, many developing countries still rush to embrace data sovereignty because data is a strategic asset to facilitate domestic and external policies.

11 BBC, “China internet: Xi Jinping calls for ‘cyber sovereignty,’” December 16,2015, https://www.bbc.com/news/world-asia-china-35109453

12 Adil Nussipov, “How China Governs Data,” April 27, 2020, https://medium.com/center-for-media-data-and-society/how-china-governs-data-ff71139b68d2

13 The Diplomat, “China’s Cybersecurity Law: What You Need to Know,” June 1, 2017, https://thediplomat.com/2017/06/chinas-cybersecurity-law-what-you-need-to-know/

14 Yuxi Wei, “Chinese Data Localization Law: Comprehensive but Ambiguous,” February 7, 2018, https://jsis.washington.edu/news/chinese-data-localization-law-comprehensive-ambiguous/

15 Ibid.

16 Adil Nussipov, “How China Governs Data,” April 27, 2020, https://medium.com/center-for-media-data-and-society/how-china-governs-data-ff71139b68d2

17 South China Morning Post, “Gym punished for improperly collecting facial data of 20,000 members,” May 15, 2020, https://www.scmp.com/abacus/news-bites/article/3084627/gym-punished-improperly-collecting-facial-data-20000-members

18 Adil Nussipov, “How China Governs Data,” April 27, 2020, https://medium.com/center-for-media-data-and-society/how-china-governs-data-ff71139b68d2

19 U.S. Department of Justice, “Promoting Public Safety, Privacy, and the Rule of Law Around the World: The Purpose and Impact of the CLOUD Act,” April 2019, https://www.justice.gov/opa/press-release/file/1153446/download

20 Ron Cheng, “Seizing Data Overseas from Foreign Internet Companies under the CLOUD Act,” May 29, 2018, https://www.forbes.com/sites/roncheng/2018/05/29/seizing-data-overseas-from-foreign-internet-companies-under-the-cloud-act/#17a4278e16c0

21 Some legal experts argue that China may have quietly established extraterritorial access to data, for example, see https://www.lawfareblog.com/did-china-quietly-authorize-law-enforcement-access-data-anywhere-world

22 See the English translation of the National Intelligence Law of the People’s Republic here https://cs.brown.edu/courses/csci1800/sources/2017_PRC_NationalIntelligenceLaw.pdf

23 For example, Reuters, “China’s intelligence law looms over EU 5G safeguards: official,” July 19, 2019, https://www.reuters.com/article/us-eu-huawei-tech/chinas-intelligence-law-looms-over-eu-5g-safeguards-official-idUSKCN1UE18I

24 VOA, “CCTV: iPhone a Threat to National Security,” July 12, 2014, https://www.voanews.com/east-asia/cctv-iphone-threat-national-security

25 BBC, “Apple hits back at China over iPhone privacy claims,” July 14, 2014, https://www.bbc.com/news/technology-28292378

26 Reuters, “China adopts cyber security law in face of overseas opposition,” November 6, 2016, https://www.reuters.com/article/us-china-parliament-cyber-idUSKBN132049

27 Reuters, “Apple moves to store iCloud keys in China, raising human rights fears,” February 24, 2018, https://www.reuters.com/article/us-china-apple-icloud-insight/apple-moves-to-store-icloud-keys-in-china-raising-human-rights-fears-idUSKCN1G8060

28 CNN, “Apple is under fire for moving iCloud data to China,” February 28, 2018, https://money.cnn.com/2018/02/28/technology/apple-icloud-data-china/index.html

29 Bloomberg, “BlackRock’s Wiseman Says Beijing Bike Rentals Show Data Is King,” October 30, 2017, https://www.bloombergquint.com/markets/blackrock-s-wiseman-says-beijing-bike-rentals-show-data-is-king

30 CNBC, “French watchdog slaps Google with $57 million fine under new EU law,” Jan 21, 2019, https://www.cnbc.com/2019/01/21/french-watchdog-slaps-google-with-57-million-fine-under-new-eu-law.html

31 Alexander Hanff, “China’s Surveillance & Social Credit system alive & kicking in Berlin…,” December 5, 2018, https://medium.com/@a.hanff/chinas-surveillance-social-credit-system-alive-kicking-in-berlin-6c2b3b10b197

32 The Telegraph, “Bike-sharing start-up Mobike close to management buyout in Europe,” April 10, 2019, https://www.chinaknowledge.com/News/DetailNews/86227/mobike-europe

33 The Verge, “Russia lifts its ban on the Telegram messenger app,” June 18, 2020, https://www.theverge.com/2020/6/18/21295535/russia-telegram-ban-lifted-security

34 For example, Apple’s 2019 transparency report can be found here https://www.apple.com/legal/transparency/pdf/requests-2019-H1-en.pdf. Facebook reports statistics of government data requests here https://transparency.facebook.com/government-data-requests, and Google’s report is here https://transparencyreport.google.com/user-data/overview?hl=en.

35 Pearson (1997) and Dickson (2003) found that private entrepreneurs are not regime challengers, despite having little political representation. Tsai (2006) argued that, when formal institutions are too rigid, private businesses use creative informal ways to bypass the restrictions of formal institutions.

36 In the late stage of this project, I discovered that the term had been used previously by other scholars in different contexts, including China’s integration into the global economic system (Steinfeld 2010), the study of comparative capitalism (Allen 2013), and elite-mass relations in Russia (Polishchuk 2013). I use the term in a distinctly different way from the prior literature. See Liu (2018) for more details.

37 Alibaba and Tencent once refused to submit their customer loan data to Baihang, a credit scoring business launched by the Chinese central bank. See Financial times, “Alibaba and Tencent refuse to hand loans data to Beijing,” September 18, 2019, https://www.ft.com/content/93451b98-da12-11e9-8f9b-77216ebe1f17

38 The Wall Street Journal, “State Support Helped Fuel Huawei’s Global Rise,” December 25, 2019, https://www.wsj.com/articles/state-support-helped-fuel-huaweis-global-rise-11577280736

39 Other Chinese social media platforms such as WeChat and Weibo have struggled to win overseas users.

40 South China Morning Post, “Why is TikTok such an online success? Ask Drake, Jane Fonda and its 800 million other monthly users,” April 10, 2020, https://www.scmp.com/lifestyle/entertainment/article/3079011/why-tiktok-such-online-success-ask-drake-jane-fonda-and-its

41 Reuters, “Exclusive: U.S. opens national security investigation into TikTok – sources,” November 1, 2019, https://www.reuters.com/article/us-tiktok-cfius-exclusive/exclusive-u-s-opens-national-security-investigation-into-tiktok-sources-idUSKBN1XB4IL

42 See the open letter at https://www.cotton.senate.gov/?p=press_release&id=1239

43 CNBC, “US opens national security investigation into TikTok,” November 1, 2019, https://www.cnbc.com/2019/11/01/us-to-investigate-tiktok-over-national-security-concerns-sources-say.html

44 Business Insider, “US government agencies are banning TikTok, the social media app teens are obsessed with, over cybersecurity fears—here’s the full list,” February 25, 2020, https://www.businessinsider.com/us-government-agencies-have-banned-tiktok-app-2020-2

45 For example, India also banned nearly 60 Chinese apps, including TikTok, in June 2020.

46 See the open letter at https://www.cotton.senate.gov/?p=press_release&id=1239

47 See TikTok’s statement at https://newsroom.tiktok.com/en-us/statement-on-tiktoks-content-moderation-and-data-security-practices

48 NBC News, “TikTok and China come under scrutiny in congressional hearing,” November 5, 2019, https://www.nbcnews.com/politics/congress/hawley-takes-aim-tiktok-china-congressional-hearing-n1076586

49 See the open letter at https://www.cotton.senate.gov/?p=press_release&id=1239

50 Techcrunch, “TikTok explains its ban on political advertising,” October 3, 2019, https://techcrunch.com/2019/10/03/tiktok-explains-its-ban-on-political-advertising/

51 The Wall Street Journal, “TikTok Searches for Global Headquarters Outside of China,” December 23, 2019, https://www.wsj.com/articles/tiktok-searches-for-global-headquarters-outside-of-china-11577097150

52 The Wall Street Journal, “TikTok to Stop Using China-Based Moderators to Monitor Overseas Content,” March 15, 2020, https://www.wsj.com/articles/tiktok-to-stop-using-china-based-moderators-to-monitor-overseas-content-11584300597

53 Pingwest, “Exclusive: ByteDance Cuts Domestic Engineers’ Data Access to TikTok, Other Overseas Products,” June 7, 2020, https://en.pingwest.com/a/6875

54 See the executive order at https://www.whitehouse.gov/presidential-actions/executive-order-addressing-threat-posed-tiktok/

55 See the executive order at https://www.whitehouse.gov/presidential-actions/order-regarding-acquisition-musical-ly-bytedance-ltd/

56 For example, the issue has been discussed at the Sixth IMF Statistical Forum, see https://www.imf.org/en/News/Seminars/Conferences/2018/04/06/6th-statistics-forum
